# Parental subjective wellbeing during the COVID-19 lockdown: Evidence from the epicenter of a pandemic crisis

**DOI:** 10.1192/j.eurpsy.2021.1759

**Published:** 2021-08-13

**Authors:** E. Biffi, A. Pepe

**Affiliations:** “r.massa” Department Of Education And Social Science, Milano-Bicocca Univeristy, Milano, Italy

**Keywords:** subjective wellbeing, COVID-19, lockdown, quantitative research

## Abstract

**Introduction:**

The Coronavirus disease (COVID-19) health emergency has led national states to adopt severe actions forcing many people to cope with new and unexpected challenges. Those constraints risked to jeopardized the mental health and subjective wellbeing (SWB) of individuals.

**Objectives:**

The present cross-sectional quantitative study explored whether and to what extent psychological and social aspects were determinants of parental SWB as outcome variable during the COVID-19 lockdown.

**Methods:**

The sample was composed of 304 Italian parents (93% female, mean age 41.5, 91% from Lombardy). Data were gathered through Computer Assisted Web Interview (CAWI) four weeks after the beginning of the national lockdown. World Health Organization (WHO) wellbeing scale along with other self-reported measures of social support, feelings of abandonment, feeling of being equipped and adequacy of living spaces were administered. Data were analyzed by hierarchical regression models (Ethics Committee of Milano-Bicocca University approval N.0034537/20)

**Results:**

According to the WHO cutoff score, 37.7% of parents reported low well-being levels. The regression model (F=11.2, p<.001) suggested that the feeling of abandonment and the feeling of being equipped were the most important contributors to SWB. Other statistically significant (but with lower effect sizes) variables were the support received by the partner and the adequacy of living spaces.

**Conclusions:**

The findings bear out the pivotal importance of subjective states (such as feelings of abandonment or perceptions of being poorly equipped) in relation to the levels of parental SWB during the COVID-19 lockdown. Implications for planning psychological interventions aimed at strengthening personal resources to face the emergency are discussed.

**Disclosure:**

No significant relationships.

